# Floating Hospital on the River Tyne

**Published:** 1887-08-13

**Authors:** Drina Abon


### FLOATING HOSPITAL ON THE RIVER TYNE.

It seems a strange way of prefacing a few remarks on a hospital to talk of its being launched, but such was the ceremony that I witnessed with regard to the particular hospital I am writing of. A floating village—I can liken it to nothing else—this home for sick seamen that rocks on the waters of the Northern river. Built to the order of the Tyne Port Sanitary Authority, this most unique structure is as perfect in every detail as the most careful erection and ingenious thought could make it. The clever engineer, Mr. W. G. Laws, is to be most heartily congratulated on his successful design, the builders (Messrs. Wood and Skinner, of Bill Quay). on having so admirably followed the plan of construction, and last, but certainly foremost in the future history of the hospital, the sailors, who, however the misfortune be deplored which leads them to require such a place, are yet to be thought happy in finding such a true harbour of refuge when overtaken by sickness. It is built upon ten cylindrical iron pontoons or floats, with hemispherical ends, each seventy feet long and six feet in diameter, resembling huge boilers, and with fourteen feet and a half of space between, measuring from centre to centre. The floating power of each is about 540 tons, and the weight of the whole complete hospital in working order about 400 tons, thus leaving 140 tons of spare buoyancy. Each pontoon is detachable, and can be removed at will for cleaning or painting, and can also be revolved in its place without removal. Internally they are reached by man-holes, placed to correspond with trap-doors in the main or platform deck. Upon each pontoon are seven "saddles," supporting a strong framework of iron, consisting of longitudinal rolled girders, fastened together by diagonal T-irons. A deck of creosoted timber is carried upon them, and on this deck or platform the hospital is erected. The deck is three inches thick, and is surrounded by a timber handrail. The platform is about 50 yards long by 25 yards wide, and thus makes a splendid promenade for the convalescent patients.

The hospital itself consists of three buildings, six smaller structures, and a mortuary. The main buildings are each 65 feet long, 23½ feet wide, and 20 feet high. Each of these are divided into two wards, one to contain six and the other four beds. The wards are built of timber, roofed with zinc, and lined with pitch-pine, varnished. The ceilings are also of pitch-pine, and above them are the cisterns for the supply of water. The drinking water is pumped in from the harbour water-boats, and that for washing purposes from the river.

In each ward there is a central shaft through the roof, fitted with a patent ventilator for carrying off the vitiated air, and under the floor of each apartment there is an air-space of about ten inches, which will secure a constant circulation of fresh air. Between the surface of the river and the platform there will be a perfectly free current of pure air; whilst the rise and fall of the tides, and the current produced by the spaces between the pontoons, will prevent the possibility of any impurity existing beneath the hospital. The three main buildings are completely isolated, so that in case of two, or even more, infectious diseases raging on board at the same time, there need be no communication between them. The windows of the blocks facing each other may have to be closed up, as a fear of the infection being blown across from one to the other is entertained. There is an entrance to each building from the deck. The wards are fitted up with every necessary appliance; between each of the two there is a nurse's room, with glazed doors on either side, so that a view of each is commanded. One bath-room does duty for the two wards, and here may be seen the latest improvements and efforts to combine the greatest comfort with the least pain. A bountiful supply of hot and cold water and roller-baths, so that the patient can be lifted straight from his bed to the bath at its side, renders the work of nursing easy both to sufferer and nurse. Indeed, from the moment he is put on board of this floating asylum everything that can be is done to mitigate pain and spare trouble. A crane, or pair of davits, is used on deck to swing the patient on board, thus avoiding all the shaking and ofttimes injury sustained in carrying sick people from the wharf.

An administrative block is a feature in the future design of the hospital, to contain a caretaker's house, doctors' rooms, and extra nurses' accommodation. Pending this, the floating power is balanced by a long box, containing ballast, placed on the left side of the platform. The building and its fittings are each in their way admirable. It only remains for good physicians and careful nurses to make the floating hospital a real boon to poor Jack, and for those Samaritans, fortunately always to be found, who make it their pleasure to brighten as much as lies in their power the homes of pain. A few flowers and pictures, some books and papers, an occasional gift of some costly, though none the less necessary, chair or other appliance, delicacies from the hot-house or larder, and, if it be possible, a few cheering words and smiles,—these are the shafts of sunlight that illumine the dark hours passed in a hospital ward. They are part of the daily life of most city hospitals; I trust they will soon be part of that on board of the sailors' temporary home. Knowing the "Blue Jackets" as I know them, I feel quite sure that such acts of kindness will be as truly appreciated by them as by any other class of sufferers. Rough, fearless, and often coarse as they are, there is yet much that is good and worthy of all respect in the British seamen. One thing I have never found amongst them, and that is ingratitude for any kindness shown them in times of trouble; and doubtless the truth of this will be seen when the wards of the new floating hospital on the Tyne are opened to the men for whom they have been designed.

DRINA ABON.

### PREACHING AND PRACTISING.

The distinguished head (a physician) of one of our charitable institutions, in the recent absence of the chaplain, conducted the usual Sunday service; and in his daily report wrote, "I preached at eleven, and at twelve practised."

